# Sildenafil Positivity and Chronic Cardiac Pathology in Men Who Died of Ischemic Heart Disease: An Age- and Sex-Matched Postmortem Case–Control Study

**DOI:** 10.3390/diagnostics16152400

**Published:** 2026-07-30

**Authors:** Alper Özkök, Özden Seçkin, Bekir Dinçer, Sait Özsoy, Taner Akar

**Affiliations:** 1 Department of Forensic Medicine, Faculty of Medicine, Gazi University, 06500 Ankara, Turkey; tanerakar@yahoo.com; 2 Department of Cardiology, Faculty of Medicine, Gazi University, 06500 Ankara, Turkey; ozden-seckin@hotmail.com; 3 Department of Forensic Medicine, Faculty of Medicine, Samsun University, 55080 Samsun, Turkey; bekir.dincer@samsun.edu.tr; 4 Department of Forensic Medicine, Health Sciences University, 06018 Ankara, Turkey; ozsoysmd@gmail.com

**Keywords:** sildenafil citrate, death, sudden, cardiac, forensic autopsy, coronary artery calcification, erectile dysfunction, phosphodiesterase 5 inhibitors

## Abstract

**Background:** Erectile dysfunction and cardiovascular disease share common pathophysiological mechanisms, yet little is known about the postmortem cardiac profile of men with detectable sildenafil use. This study investigated whether postmortem sildenafil positivity is associated with acute or chronic cardiac pathological findings in men who died of ischemic heart disease. **Methods:** In a sample of 154 male decedents who died of ischemic heart disease, postmortem cardiac and toxicological findings in 77 sildenafil-positive cases were compared with those of age-matched controls. Heart weight z-scores were calculated based on 27,645 reference autopsies. Multivariable logistic regression analyses were performed to identify independent associations. **Results:** No significant differences were observed between groups regarding acute ischemic pathologies. However, chronic cardiac findings were more prevalent in the sildenafil-positive group, including coronary artery disease (96.1% vs. 85.7%; *p* = 0.025), congestive heart failure (40.3% vs. 24.7%; *p* = 0.039), prior coronary artery bypass grafting (24.7% vs. 11.7%; *p* = 0.037), increased heart weight (547.7 gr vs. 507.5 gr; *p* = 0.037) and z-score (+1.63 vs. 1.08; *p* = 0.040), and coronary calcification (88.3% vs. 74.0%; *p* = 0.023). After adjustment for cardiovascular comorbidities, only coronary calcification remained independently associated (adjusted OR: 2.586; 95% CI: 1.076–6.215; *p* = 0.034). **Conclusions:** Sildenafil-positive cases showed no excess of acute ischemic pathology. However, sildenafil-positive cases had a greater burden of chronic cardiac pathology, particularly coronary calcification. These findings are consistent with the proposed role of coronary artery calcification as a surrogate marker of erectile dysfunction and cardiovascular disease. They also underscore the importance of cardiovascular risk assessment in sildenafil users and support a cautious approach in individuals with poor cardiac reserve.

## 1. Introduction

Sudden cardiac death (SCD) remains a leading cause of mortality worldwide, with ischemic heart disease accounting for the vast majority of cases, especially among older males with underlying comorbidities [1]. While erectile dysfunction (ED) is a common condition in this population and shares many pathophysiological mechanisms with cardiovascular disease (CVD), the cardiovascular safety of phosphodiesterase type 5 inhibitors (PDE5i), such as sildenafil, continues to raise clinical and forensic concerns [2].

Although randomized controlled trials and observational data have not demonstrated a direct proarrhythmic or ischemia-inducing effect of sildenafil in appropriately selected patients, real-world scenarios often involve polypharmacy, undiagnosed cardiac pathology, and unsupervised medication use, complicating risk stratification [3,4]. Autopsy-based evaluations offer a unique opportunity to assess the structural and pathological cardiac profiles of sildenafil users in fatal outcomes, particularly in the context of sudden deaths [1].

This study aims to explore whether postmortem sildenafil positivity is associated with acute or chronic cardiac pathological findings among men who died of ischemic heart disease. By employing a retrospective age- and sex-matched case–control design and comprehensive forensic autopsy protocols aligned with international guidelines, we seek to clarify whether postmortem sildenafil positivity may reflect underlying cardiac vulnerability or act as a direct precipitant of cardiac death.

## 2. Materials and Methods

### 2.1. Study Design and Case Selection

**Figure 1:**
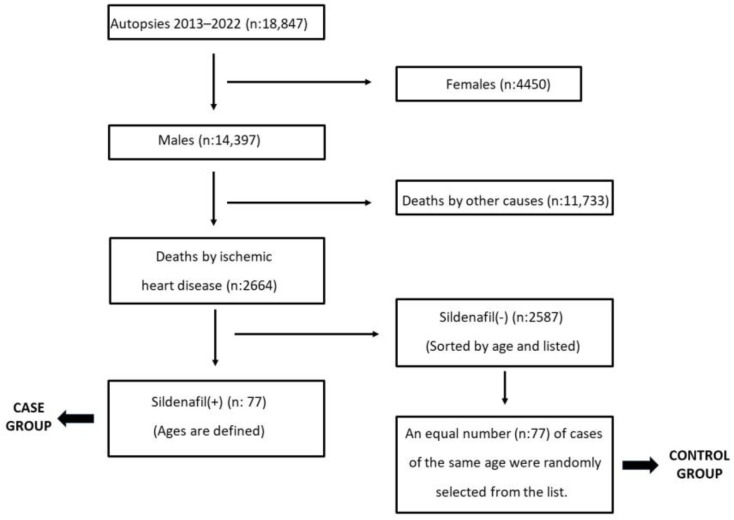
Study design and selection of the case and control groups.

This retrospective case–control study examined autopsy records collected between 2013 and 2022. Male decedents whose toxicological analyses revealed the presence of sildenafil in their blood samples and whose cause of death was determined to be ischemic heart disease were identified as the case group. To establish the study population, all male cases from the specified period were first filtered based on causes of death classified under ischemic heart disease categories [5]. Of 2664 such cases, 77 individuals with detectable sildenafil in their toxicology reports were selected as cases. The remaining 2587 were listed by age using an Excel spreadsheet. For each sildenafil-positive case, one age-matched sildenafil-negative male decedent was randomly selected as a control from this list. The methodological framework and the group selection process are illustrated in Figure 1. The study was granted by the Education and Scientific Research Commission of the Council of Forensic Medicine, Türkiye (2023/392), and Helsinki Declaration rules were followed to conduct this study. Since the study was retrospective in nature and postmortem, informed consent was not applicable.

### 2.2. Data Collection and Autopsy Procedures

Relevant data were retrieved from premortem medical records, prosecutors’ reports (covering postmortem scene investigations, witness and family interviews), final emergency department records (if applicable), and comprehensive autopsy findings, including external and internal examinations, histopathologic and toxicologic analyses. All autopsies were conducted at a high-volume forensic center located in Ankara, Türkiye, which performs approximately 2000 autopsies annually. In accordance with the Turkish Criminal Procedure Code [6], each autopsy was conducted by two board-certified forensic medicine specialists. All procedures followed the principles of the European Council of Forensic and Legal Medicine (ECLM) and reflected contemporary guidelines for cardiac examination [7,8].

### 2.3. Clinical and Pathological Variables

Diagnoses of hypertension (HT), hyperlipidemia (HL), diabetes mellitus (DM), chronic coronary artery disease (CCAD), congestive heart failure (CHF), and other systemic diseases associated with cardiovascular risk were identified and recorded from medical records and prosecutors’ reports. As pulmonary arterial hypertension represents an established indication for sildenafil use other than erectile dysfunction, it was specifically considered during file review.

Because clinical diagnoses of erectile dysfunction were not systematically available in the medical records, sildenafil positivity was used solely as a toxicological exposure variable and not as confirmation of erectile dysfunction or chronic sildenafil treatment.

The category of “other systemic diseases associated with cardiovascular risk” encompassed only conditions well-established in the literature as contributing to cardiovascular risk beyond traditional major risk factors, such as chronic kidney disease, rheumatoid arthritis, and systemic lupus erythematosus [9]. Since this category was defined as a dichotomous variable (“present/absent”), cases with multiple such diseases were still recorded only as “present,” and no further grading was applied. The total number of diseases was also recorded separately as another variable (no. of diseases).

The presence of coronary stents, coronary artery bypass grafting (CABG), the mean and maximum coronary artery stenosis levels, evidence of coronary thrombosis or ruptured plaques (acute coronary artery disease, ACAD), coronary calcification, heart weight, myocardial structural condition, presence of hypertrophic fibers, and the estimated duration of myocardial ischemia were identified and recorded from the autopsy and histopathology reports.

### 2.4. Diagnostic Criteria and Histological Evaluation

For heart weight assessment, one outlier above 900 g was excluded from each group to ensure normal distribution. Whether the hearts were overweight (normal or >1 SD) was calculated using an online calculator “http://lundforensicmedicine.com (accessed on 5 September 2025)”, standardized with data from 27,645 reference autopsies, and adjusted for sex, height, weight, and age. Heart weight exact standard deviation (z-score) for each case was calculated using the formulas provided in the same study, and comparisons were made between the two groups [10]. CCAD was diagnosed premortem or by autopsy evidence of at least 50% luminal stenosis, coronary stenting, or CABG [11,12,13].

Coronary artery calcification (CAC) was initially evaluated macroscopically during the autopsy by forensic medicine specialists and subsequently confirmed by histopathological examination performed by a pathologist. Macroscopic examination included visual inspection and digital palpation of the coronary arteries for the presence of hardening, crepitus, and chalk-white or grayish calcific deposits on the luminal surface and within the arterial wall. For histopathological assessment, cross-sections of the coronary arteries were obtained at standardized intervals and processed using routine hematoxylin and eosin staining. Calcification was identified as basophilic, amorphous deposits within the intimal or medial layers on hematoxylin and eosin-stained sections. Based on the integrated findings from macroscopic and histopathological evaluations, CAC was dichotomously classified as present or absent. Since postmortem blood toxicology analyses were completed after the macroscopic and microscopic examinations, CAC assessments were blind to sildenafil positivity. Average coronary stenosis was calculated by averaging occlusion rates across all major vessels. Maximum stenosis was determined by identifying the most severely occluded coronary artery in each case.

Myocardial findings in the histopathology reports were classified into two main categories for binary comparison. Cases with no lesions or reactive fibrosis (perivascular and interstitial) were grouped into one category (no lesion/mild), while cases with replacement fibrosis (focal myocardial necrosis or more, including aneurysms) were grouped into the other category (severe) [14,15]. When multiple findings were present, classification was based on the most severe lesion. The duration of myocardial ischemia was estimated histologically and grouped into <24 h, 1–14 days, 2–8 weeks, or >8 weeks. In cases with multiple ischemic phases, the most recent lesion was used for classification.

### 2.5. Identifying Toxicological Variables and Case Selection

Comprehensive toxicological screening, including analysis for medical drugs (including PDE5 inhibitors), illicit substances, and ethanol, was performed using femoral venous blood collected routinely at autopsy. In the toxicological results, PDE5 inhibitor findings were categorized as sildenafil-only, other PDE5 inhibitors-only, or concurrent detection of agents. Four cases in which tadalafil was detected, four cases in which both sildenafil and tadalafil were detected, and one case in which sildenafil, tadalafil, and vardenafil were detected were excluded from the study. In the remaining 77 sildenafil-positive cases and 77 control cases, the following parameters were identified and recorded: Sildenafil blood level (in the case group), co-presence of contraindicated medications, presence of antihypertensive agents (including beta-blockers, alpha-blockers, diuretics, ACE inhibitors, calcium channel blockers, and angiotensin receptor blockers), anti-diabetics, anti-aggregants and coagulants, anti-arrhythmics, NSAIDs, anti-histamines, the total number of antihypertensive medications detected, as well as the presence of alcohol or illicit drugs. Quantitative blood concentrations of medications are determined when analytically and medico-legally indicated as part of routine forensic practice. Accordingly, quantitative data for other medications were available for only a subset of cases, while sildenafil concentrations were present in most cases (*n* = 68).

### 2.6. Statistical Analysis

Statistical analyses were performed using SPSS 29.0.2.0. Categorical variables were summarized as frequencies and percentages; continuous variables as mean ± SD or median (IQR), depending on distribution. Normality was assessed visually (histograms and probability plots) and with the Kolmogorov–Smirnov test. Chi-square or Fisher’s exact test was used for categorical comparisons. Student’s *t*-test was applied to normally distributed variables, and the Mann–Whitney U test to non-normal or ordinal variables.

To assess the independent association between sildenafil positivity and each cardiac pathology measure, univariable logistic regression analyses were first performed for each measure. Then, for each measure with a *p*-value < 0.20 in the univariable analyses, a separate multivariable logistic regression model was constructed. All multivariable models were adjusted for obesity, HT, HL, DM, and other systemic diseases associated with cardiovascular risk, all of which were categorized as binary variables (present/absent). Since the sildenafil (+) and sildenafil (−) groups were matched for age and sex, these variables were not included in the multivariable models (ORs with %95 CI and *p* < 0.05).

## 3. Results

### 3.1. Demographic and Clinical Characteristics

**Figure 2:**
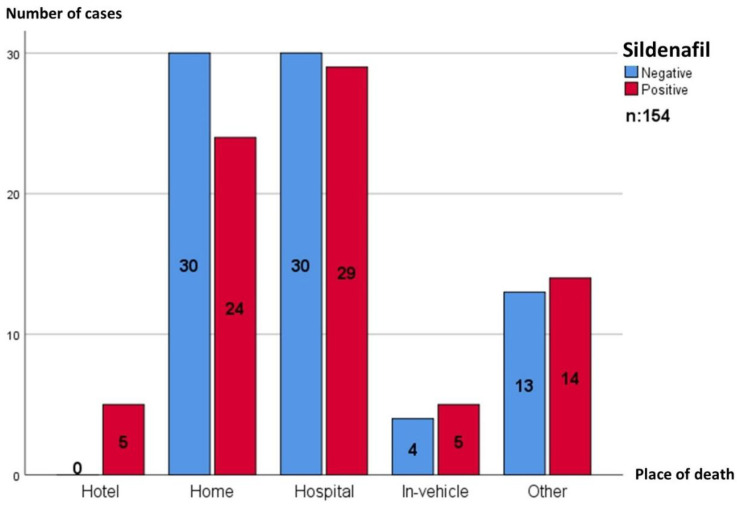
Place of death of the cases.

The distribution of places of death was nearly identical in both groups, except that five of the sildenafil-positive deaths occurred in hotel rooms (Figure 2).

No statistically significant difference was found between the groups in terms of BMI (28.3 ± 3.65 vs. 28.7 ± 4.36; *p* = 0.599) or obesity prevalence (33.8% in both groups; *p* > 0.999). Although HT, HL, and DM were more frequently observed in the sildenafil-positive group, these differences did not reach statistical significance. The number of cases with other systemic diseases known to increase cardiovascular risk was similar across both groups. However, the total disease burden approached significance, being higher in the sildenafil-positive group (median: 4 [IQR 3–6] vs. 3 [IQR 2–5]; *p* = 0.058) (Table 1).

### 3.2. Cardiac Findings: Acute vs. Chronic Pathology

**Figure 3:**
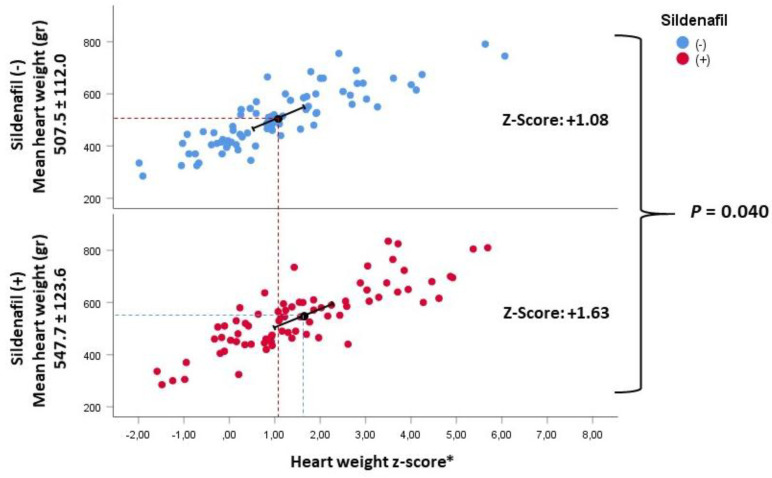
Comparison of standardized heart weight z-scores between sildenafil-positive and -negative cases. * The z-score represents the standard deviations from the expected mean heart weight given sex, age, height, and body weight.

No significant difference was found between groups regarding acute coronary artery disease (ACAD; *p* = 0.258) or myocardial ischemia of <24 h duration (*p* = 0.367). In contrast, findings related to chronic cardiac pathology were more prevalent in almost every measure in the sildenafil-positive group with significant differences for the prevalence of chronic coronary artery disease (CCAD; *p* = 0.025), congestive heart failure (CHF; *p* = 0.039), coronary artery bypass grafting (CABG; *p* = 0.037), mean heart weight (*p* = 0.037), and coronary calcification (*p* = 0.023). The frequency of overweight hearts (greater than 1 SD) (*p* = 0.024) and the means of heart weight z-scores (*p* = 0.040), standardized by age, gender, height, and weight, were also higher in the sildenafil-positive group (Table 1, Figure 3). However, the degree of coronary stenosis did not differ between groups for either average occlusion (*p* = 0.960) or maximum occlusion (*p* = 0.583) and the number of cases in which a coronary stent was identified at autopsy was nearly identical between the groups (*p* = 0.837) (Table 1).

Histopathologic analyses revealed no differences between the groups regarding hypertrophic myocardial fibers, severe myocardial changes, or the distribution of myocardial ischemia durations. Nevertheless, the proportion of cases with severe myocardial changes approached statistical significance (*p* = 0.064) (Table 1).

### 3.3. Toxicological Findings

The maximum measured sildenafil concentration among positive cases was 654 ng/mL. Thus, no case exhibited a toxic sildenafil level, according to the reference thresholds [16]. Besides sildenafil, at least one additional drug was identified in 49 sildenafil-positive cases and in 51 controls. These included a broad spectrum of medications ranging from antibiotics and antihistamines to antidepressants and antipsychotics, with NSAIDs being the most frequently detected.

There were no statistically significant differences between the groups in terms of the presence, type, or number of antihypertensive medications detected in blood samples. This also held true for combinations of antihypertensive agents. Alcohol was detected in 8 cases (10.4%) in the control group and 9 cases (11.7%) in the sildenafil group (*p* = 0.797). No illicit drugs were detected in either group.

### 3.4. Regression Analyses

In univariable logistic regression, the presence of CCAD (OR: 4.111; *p* = 0.036), CHF (OR: 2.057; *p* = 0.040), CABG (OR: 2.475; *p* = 0.040), average heart weight (OR: 1.003; *p* = 0.048), overweight heart (>1 SD) (OR: 2.093; *p* = 0.025), heart weight z-score (OR: 1.217; *p* = 0.046), and coronary calcification (OR: 2.651; *p* = 0.027) were significantly associated with sildenafil-positivity. However, in multivariable analyses adjusted for obesity, HT, HL, DM, and other systemic diseases associated with cardiovascular risk, only coronary calcification remained independently associated with sildenafil-positivity (adjusted OR: 2.586; *p* = 0.034) (Table 2).

## 4. Discussion

### 4.1. Overall Evaluation of the Results

In this age- and sex-matched autopsy-based case–control study, no difference was observed between sildenafil-positive and negative groups regarding acute ischemic pathology measures. On the other hand, regarding chronic cardiac pathologies, the sildenafil-positive group showed higher values in almost every variable, with significant differences for CCAD, CHF, increased heart weight, history of CABG, and coronary calcification. After adjustment for cardiovascular comorbidities, only coronary calcification remained independently associated with sildenafil positivity.

### 4.2. Sildenafil Positivity and Acute Ischemic Pathologies

This study found no evidence suggesting increased acute ischemic pathologies among sildenafil-positive cases. The prevalence of acute coronary thrombosis and of myocardial ischemia within the first 24 h was comparable in both groups, arguing against increased thrombus formation, direct pro-arrhythmic or vasospastic role of sildenafil in these deaths. However, the assessment of ischemic changes and coronary thrombosis alone cannot definitively exclude a potential contribution of sildenafil to acute ischemic cardiac pathologies. Nevertheless, despite rare anecdotal reports [17,18], data from pre-approval phase II/III trials and post-marketing surveillance have not demonstrated an increased risk of myocardial infarction, mortality, or major cardiovascular events among sildenafil users [19,20,21,22]. Furthermore, accumulating evidence from large-scale studies and meta-analyses conducted over the past decade has generally contributed to a growing consensus regarding the safety of PDE5 inhibitors in men with ED [23,24,25].

### 4.3. Sildenafil Positivity and Chronic Cardiac Pathologies

On the other hand, chronic cardiac conditions, including CCAD, CHF, prior CABG, increased heart weight, and coronary calcification, were more frequent in the sildenafil-positive group. Myocardial fibrosis, another marker of cardiac structural remodeling, tended to be more prominent in the sildenafil-positive group, also accompanied by a slightly higher prevalence of cardiovascular comorbidities. Although a diagnosis of erectile dysfunction (ED) could not be confirmed from the available records, sildenafil-positive decedents, presumed to have ED (as there was no case with a diagnosis of pulmonary arterial hypertension in the sildenafil-positive group), appeared to have a less favorable overall cardiac profile. Given that ED is a recognized marker of systemic endothelial dysfunction and shares common risk factors with CVD [25,26,27,28,29,30,31,32], the overall greater burden of cardiac pathology observed in the sildenafil-positive group is consistent with the established association between ED and CVD in the literature. Nevertheless, our findings should not be generalized to all sildenafil users, as the study population was limited to postmortem cases with ischemic heart disease.

### 4.4. Sildenafil Positivity and Coronary Artery Calcification (CAC)

In the multivariate model adjusted for cardiovascular comorbidities, the CCAD also approached statistical significance; however, only coronary calcification remained independently associated with sildenafil positivity. The ED-CAC association is supported by multiple clinical studies. Chiurlia et al. were among the first to demonstrate a 3.68-fold higher likelihood of CAC > 75th percentile in ED patients compared with controls [33]. Subsequent studies have reinforced this link. In the Multi-Ethnic Study of Atherosclerosis cohort, ED patients had a higher prevalence of CAC scores > 100 compared to men without ED (36.4% vs. 17.2%), with only CAC >100 remaining significantly associated with ED in multivariate analysis (odds ratio: 1.43; 95% CI: 1.09–1.88) [34]. In a cohort of 1119 men enrolled in an ongoing cardiac screening program, individuals with CAC scores above the 75th percentile (defined as high-risk CAC) were significantly more likely to have ED, with a 54% higher prevalence of high-risk CAC among men with ED [35]. Additionally, in a prospective study of 9150 men, measurable CAC on CT was more frequent among individuals with ED than among those without ED (79% vs. 58%, *p* < 0.001) [36]. Lastly, three separate studies from Türkiye reported a positive correlation between CAC scores and ED severity [37,38,39]. Today, contemporary guidelines recognize the CAC score as a superior predictor of CVD risk compared with other bio or imaging markers [25,40,41]. As a result, the 4th Princeton Consensus incorporated an algorithm into the evaluation of men presenting with ED, derived from the latest recommendations of the American College of Cardiology (ACC) and the American Heart Association (AHA), which includes computed tomography-based CAC scoring [25]. Although ED status could not be confirmed in our study population, the independent association between coronary calcification and sildenafil positivity provides postmortem support for previous clinical observations. Together, these findings support the proposed role of coronary artery calcification as a surrogate marker of erectile dysfunction.

### 4.5. Sexual Activity, Physical Exertion, and Timing of Death

According to ACC and AHA guidelines and the 4th Princeton Consensus, coital exertion may precipitate myocardial infarction or cardiovascular decompensation in high-risk individuals and should be avoided in patients with unstable or poorly controlled CVD until clinical stabilization is achieved. Similarly, sildenafil is also contraindicated in patients with CHF and borderline low blood pressure and borderline low volume status [17,25,42]. Although real-time functional assessments (e.g., stress testing) were not applicable in our study sample, it is plausible that sildenafil users with reduced cardiac reserve may have experienced fatal decompensation during exertion. In our matched groups, consistent with current guideline recommendations, CHF was more prevalent in the sildenafil-positive group. However, information on the timing of sildenafil administration, whether sexual activity actually occurred, and the interval between sexual intercourse and death was generally unavailable in the medical and prosecutorial records. Therefore, the higher prevalence of CHF should be interpreted cautiously; whether it reflects the hemodynamic stress of sexual activity, a potential effect of sildenafil itself, or a combination of both remains to be elucidated. In addition, sildenafil use is considered high risk in patients with myocardial ischemia of less than 2 weeks’ duration, intermediate risk in those with ischemia between 2–6 weeks, and low risk in cases exceeding 6 weeks [17,25]. However, in our study, the distribution of myocardial ischemia durations did not differ significantly between the age-matched sildenafil-positive and control groups, providing no support for this risk stratification.

### 4.6. Limitations

One limitation of this study is the inclusion of only postmortem cases, which may introduce selection bias and thereby limit generalizability to the living population. Similarly, although no diagnosis of pulmonary arterial hypertension was identified in our groups, the findings cannot be generalized to ED overall or to PDE5 inhibitors as a class. Clinical tools such as the NYHA classification and quantitative CT-based CAC scoring could not be applied or were only partially applicable. Instead, reliance on a dichotomous assessment of coronary artery calcification, rather than quantitative thresholds (e.g., CAC score > 100 or >75th percentile), represented an additional limitation. Moreover, multiple clinical and pathological variables were compared without formal adjustment for multiple testing. Therefore, secondary findings should be interpreted cautiously because some statistically significant associations may have occurred by chance.

The retrospective design imposed further constraints. Detailed clinical data, including pre-mortem functional cardiovascular status, and key confounders such as smoking, sedentary lifestyle, and alcohol habit, were not systematically recorded in the medical records. Consequently, residual confounding cannot be excluded, and part of the observed associations may reflect differences in unmeasured cardiovascular risk factors rather than sildenafil positivity itself. Finally, none of the cases had toxic levels of sildenafil, and no findings indicated concomitant use of contraindicated medications or adverse interactions with antihypertensive agents. Nevertheless, as toxicological analyses were limited to substances detectable postmortem and those included in routine postmortem panels, some rare drug interactions and the presence of rapidly eliminated or low-concentration drugs (e.g., short-acting nitrates or CYP3A4 inhibitors) may not have been detected. Furthermore, because postmortem redistribution may affect measured drug concentrations, toxicological findings should be interpreted with appropriate caution.

### 4.7. Strengths

On the other hand, the age- and sex-matched design of the study, adjustment for cardiac comorbidities, restriction to sildenafil-positive cases (excluding other PDE5 inhibitors), and inclusion of only cases in which the cause of death was sudden cardiac death due to myocardial ischemia allowed the exclusion of many potential confounders. Although retrospective in nature, the study is based on a 10-year dataset derived from systematically conducted, comprehensive, and reliable forensic autopsy reports. In addition, the postmortem setting enabled the use of direct, unmediated, high-validity and accuracy cardiac examination methods, through macroscopic and histopathological analyses, which are rarely feasible in clinical research. Thus, cardiac structures were directly assessed; coronary calcification, a key outcome of the study, was visualized macroscopically and confirmed microscopically; and heart weight was accurately determined, further supported by standardized z-scores derived from a large reference autopsy dataset.

## 5. Conclusions

This study found no evidence that postmortem sildenafil positivity is associated with acute ischemic cardiac pathology, among men who died of ischemic heart disease. Instead, sildenafil-positive decedents exhibited a greater burden of chronic cardiac pathologies, with coronary calcification remaining the only independently associated finding after adjustment. These findings are consistent with the proposed role of CAC as a surrogate marker of ED and CVD reported in the literature, and more plausibly reflect an increased cardiovascular disease burden among sildenafil-positive individuals rather than a direct adverse effect of sildenafil itself. Nevertheless, limitations regarding the postmortem design, diagnosis of erectile dysfunction, and potential drug interactions should be taken into account when interpreting the study findings. A cautious approach may be beneficial when considering sildenafil use in individuals with poor cardiac reserve or structural cardiac abnormalities, especially in the context of exertion. Further prospective clinical studies with larger cohorts are needed to better elucidate these associations.

## Figures and Tables

**Table 1:** Comparison of findings between the sildenafil-positive and sildenafil-negative groups.

	Sildenafil (−)	Sildenafil (+)	*p*-Value
Sex (male), n (%)	77 (100)	77 (100)	>0.99
Age, mean ± SD	58.77 ± 9.987	58.77 ± 9.987	>0.99
BMI, mean ± SD	28.3 ± 3.65	28.7 ± 4.36	0.599
Obesity (BMI ≥ 30), n (%)	26 (33.8)	26 (33.8)	>0.99
Hypertension, n (%)	41 (53.2)	50 (64.9)	0.140
Hyperlipidemia, n (%)	20 (26.0)	28 (36.4)	0.164
Diabetes mellitus, n (%)	19 (24.7)	27 (35.1)	0.159
CCAD, n (%)	66 (85.7)	74 (96.1)	0.025
CHF, n (%)	19 (24.7)	31 (40.3)	0.039
Other systemic diseases *, n (%)	38 (49.4)	36 (46.8)	0.747
No. of diseases, median (IQR)	3 (2–5)	4 (3–6)	0.058
Coronary stent, n (%)	14 (18.2)	15 (19.5)	0.837
CABG, n (%)	9 (11.7)	19 (24.7)	0.037
Average heart weight (gr) ± SD	507.5 ± 112.0	547.7 ± 123.6	0.037
Overweight heart (>1 SD), n (%)	34 (44.2)	48 (62.3)	0.024
Hypertrophic fibers, n (%)	43 (55.8)	52 (67.5)	0.136
Severe changes (replacement fibrosis), n (%)	58 (75.3)	67 (87.0)	0.064
Coronary calcification, n (%)	57 (74.0)	68 (88.3)	0.023
Average coronary stenosis—median (IQR)	75 (57–92)	78 (62–89)	0.960
Maximum coronary stenosis—median (IQR)	85 (70–95)	90 (77–93)	0.583
ACAD, n (%)	14 (18.2)	9 (11.7)	0.258
Myocardial ischemia duration:			
No lesion, n (%)	2 (2.6)	2 (2.6)	>0.99
<24 h, n (%)	4 (5.2)	1 (1.3)	0.367
1 day–2 weeks, n (%)	15 (19.5)	12 (15.6)	0.525
<2 weeks, n (%)	19 (24.7)	13 (16.9)	0.233
2–8 weeks, n (%)	6 (7.8)	9 (11.7)	0.415
>2 months, n (%)	50 (64.9)	53 (68.8)	0.607
Medicine (other than Sildenafil), n (%)	51 (66.2)	49 (63.6)	0.736
Anti-hypertensives, n (%)	20 (26.0)	23 (29.9)	0.590
Beta-blockers, n (%)	12 (15.6)	14 (18.2)	0.667
Alfa-blockers, n (%)	0 (0.0)	1 (1.3)	>0.99
Diuretics, n (%)	8 (10.4)	4 (5.2)	0.229
ACE inhibitors, n (%)	4 (5.2)	3 (3.9)	>0.99
Ca channel blockers, n (%)	4 (5.2)	4 (5.2)	>0.99
Angiotensin receptor blockers, n (%)	3 (3.9)	2 (2.6)	>0.99
Multiple anti-hypertensive use, n (%)	9 (11.7)	5 (6.5)	0.262
Anti-diabetics, n (%)	11 (14.3)	11 (14.3)	>0.99
Anti-aggregants/coagulants, n (%)	10 (13.0)	12 (15.6)	0.645
Anti-arrhythmics, n (%)	13 (16.9)	18 (23.4)	0.315
NSAIDs, n (%)	21 (27.3)	22 (28.6)	0.857
Anti-histamines, n (%)	8 (10.4)	10 (13.0)	0.616
Alcohol, n (%)	8 (10.4)	9 (11.7)	0.797
Illegal drug, n (%)	0	0	-

* Other systemic diseases associated with cardiovascular risk.

**Table 2:** Regression analysis of predictors for sildenafil use in autopsy cases that died from cardiac ischemic causes.

	Sex- and Age-Matched, Unadjusted, OR (%95 CI)	*p*-Value	Sex- and Age-Matched, Adjusted *, OR (%95 CI)	*p*-Value
CCAD	4.111 (1.099–15.375)	0.036	3.550 (0.917–13.742)	0.067
CHF	2.057 (1.032–4.101)	0.040	1.742 (0.818–3.712)	0.150
Coronary stent	1.089 (0.485–2.443)	0.837		
CABG	2.475 (1.040–5.890)	0.040	2.146 (0.840–5.478)	0.110
Average heart weight	1.003 (1.000–1.005)	0.048	1.002 (0.999–1.005)	0.174
Overweight heart >1 SD	2.093 (1.099–3.986)	0.025	1.854 (0.932–3.687)	0.079
Heart weight z-score	1.217 (1.004–1.476)	0.046	1.165 (0.939–1.445)	0.165
Coronary calcification	2.651 (1.120–6.277)	0.027	2.586 (1.076–6.215)	0.034
Coronary stenosis av.	1.007 (0.993–1.022)	0.305		
Coronary stenosis max.	1.015 (0.999–1.031)	0.067	1.013 (0.997–1.029)	0.114
ACAD	0.596 (0.241–1.472)	0.262		
Hypertrophic fibers	1.645 (0.854–3.168)	0.137	1.463 (0.732–2.924)	0.282
Myocardial fibrosis	2.195 (0.945–5.097)	0.067	1.825 (0.749–4.445)	0.185

* Adjusted for: Obesity, HT, HL, DM, and other systemic diseases associated with cardiovascular risk. CCAD: Chronic Coronary Artery Disease, CHF: Congestive Heart Failure, CABG: Coronary Artery Bypass Graft.

## Data Availability

The data and materials can be shared by the corresponding author upon reasonable request. Raw data cannot be made publicly available due to legal restrictions.

## Abbreviations

The following abbreviations are used in this manuscript:

HT	Hypertension
HL	Hyperlipidemia
DM	Diabetes Mellitus
ACAD	Acute Coronary Artery Disease
CCAD	Chronic Coronary Artery Disease
CHF	Congestive Heart Failure
CABG	Coronary Artery Bypass Graft
CVD	Cardiovascular Disease
ED	Erectile Dysfunction
CAC	Coronary Artery Calcification
